# Supplementary material for: Quantifying and categorising the animal welfare impacts caused by biological invasions
Source: Nat Commun. 2026 May 5;17:3899. doi: 10.1038/s41467-026-72154-9 (PMC13144615; doi:10.1038/s41467-026-72154-9)
Supplement: Supplementary file 1 — Supplementary Information [file 41467_2026_72154_MOESM1_ESM.pdf]

Quantifying and categorising the animal welfare impacts caused by biological invasions

Thomas Evans and Michael Mendl

Supplementary Information

Supplementary Note 1

AWICIS – guidelines for application --- 02 – 20

Supplementary Note 2

Testing the framework -- 21 – 23

Table S5: Results of assessments carried out by three assessors to test the AWICIS framework and assessment template --- 23

Supplementary Note 3

Tables S6 – S11: Contingency table tests --- 24 – 25

References --- 26 – 29

Supplementary Note 1

Animal Welfare Impact Classification for Invasion Science (AWICIS) – guidelines for application

1. Scope -- 04 – 05

1.1 Defining animal welfare --- 04

1.2 Animal welfare vs species survival --- 04

1.3 What types of animals can be assessed? -- 04

1.4 Individual animals --- 04

1.5 Native and introduced animals; wild and domesticated animals ---------------------------- 05

1.6 Animal welfare impacts associated with the management of biological invasions ------ 05

Figure S1: Key aspects of the AWICIS framework, including an impact example -- 06

2. Categorising impacts -- 07 – 09

2.1 Impact mechanisms (adopted from the EICAT framework[1], with amendments) ----- 07

Table S1: AWICIS – impact mechanisms --- 07

Figure S2: AWICIS – impact mechanism descriptions and examples ----------------- 08

2.2 Links with domains of animal welfare (as described in the *'2020 Five Domains Model for Animal Welfare Assessment and Monitoring'*[2]) -- 09

3. Quantifying impacts -- 09 – 17

3.1 Identifying relative changes to the welfare of an individual ---------------------------------- 09

3.1.1 Impacts on native animals --- 09

3.1.2 Impacts on introduced animals -- 09

3.1.3 Prevalence -- 10

3.2 Short-term vs prolonged impacts --- 11

3.3 Taxonomic extent of assessments -- 11

3.4 Impact severity categories -- 11

Figure S3. AWICIS – the five impact severity categories --------------------------------- 12

Table S2. AWICIS – impact severity categories -- 13

3.5 Establishing impact severity --- 13

Table S3. AWICIS – identifying category (iv) and (v) impacts --------------------------- 14 – 15

3.6 Assessing diverse impacts on an individual animal -- 16

3.7 Comparing impact severity across affected individuals -------------------------------------- 16

3.8 Consideration of welfare impacts that are not caused by biological invasions ----------- 16

3.9 Numbers of affected individuals -- 16 – 17

4. Evidence of impacts -- 17 – 18

4.1 Impacts occurring under natural conditions -- 17

4.2 Physical, behavioural and physiological indicators -- 17 – 18

Table S4. AWICIS – indicators of animal welfare impacts ------------------------------- 18

4.3 Describing the full causal chain associated with an impact --------------------------------- 18

5. Assessment scale --- 19

6. Assessment confidence -- 19 – 20

7. AWICIS assessment template -- 20

1. Scope

1.1 Defining animal welfare

AWICIS adopts the following broad definition of animal welfare, which is taken from the World Organisation for Animal Health:

> 'the physical and mental state of an animal in relation to the conditions in which it lives and dies'[3].

1.2 Animal welfare vs species survival

AWICIS assesses the impacts of biological invasions on the welfare of animals. Other frameworks have been published which assess different types of impacts that are caused by biological invasions. These frameworks include the Environmental Impact Classification for Alien Taxa (EICAT)[1,4], which assesses the impacts of introduced species on native biodiversity. Hence, EICAT assesses impacts that affect the survival of native species, including their ability to reproduce and maintain viable populations. AWICIS does not assess impacts on species survival.

1.3 What types of animals can be assessed?

Sentience is 'the ability to have physical and emotional experiences, which matter to the animal, and which can be positive and negative'[5]. AWICIS can be used to assess the impacts of biological invasions on the welfare (physical and mental state) of both vertebrate and invertebrate species. However, when considering mental states to be the key determinant of welfare, inferences about such states can be made most strongly for animals that are protected under the Animal Welfare (Sentience) Act 2022[6] and assumed, at least in UK legislation, to be sentient. Currently, these are non-human vertebrates, cephalopod molluscs and decapod crustaceans.

1.4 Individual animals

AWICIS assesses welfare impacts on individual animals. 'Individuals' are considered to be notional typical representatives of a species of interest who, on average, are likely to experience an identified welfare impact in broadly the same manner – clearly actual individuals will, due to individual and random factors, vary in exactly how their welfare is affected.

1.5 Native and introduced animals; wild and domesticated animals

AWICIS assesses welfare impacts on native and introduced animals, both wild and domesticated. Thus, under AWICIS interactions causing animal welfare impacts may include introduced species affecting the welfare of an individual native or introduced animal, and native species affecting the welfare of an individual introduced animal or (perhaps more rarely) individual native animal.

Interactions between two animals arising from a biological invasion may adversely affect them both, and in different ways. For example, introduced cane toads (*Rhinella marina*) in Australia that are preyed on by native northern quolls (*Dasyurus hallucatus*) are injured and killed, whilst the quolls are poisoned because the toads are toxic[7]. Welfare impacts on both interacting animals can be assessed using AWICIS.

1.6 Animal welfare impacts associated with the management of biological invasions

The management of biological invasions can result in adverse welfare impacts on animals – particularly introduced animals that are controlled (e.g., through trapping or shooting) because they have, or are perceived to have damaging impacts on native biodiversity[8]. However, AWICIS does not assess impacts resulting from interactions between humans and animals, and hence cannot be used to assess impacts on introduced species associated with their management.

Figure S1 illustrates the links between the main aspects of the AWICIS framework as described in these guidelines.

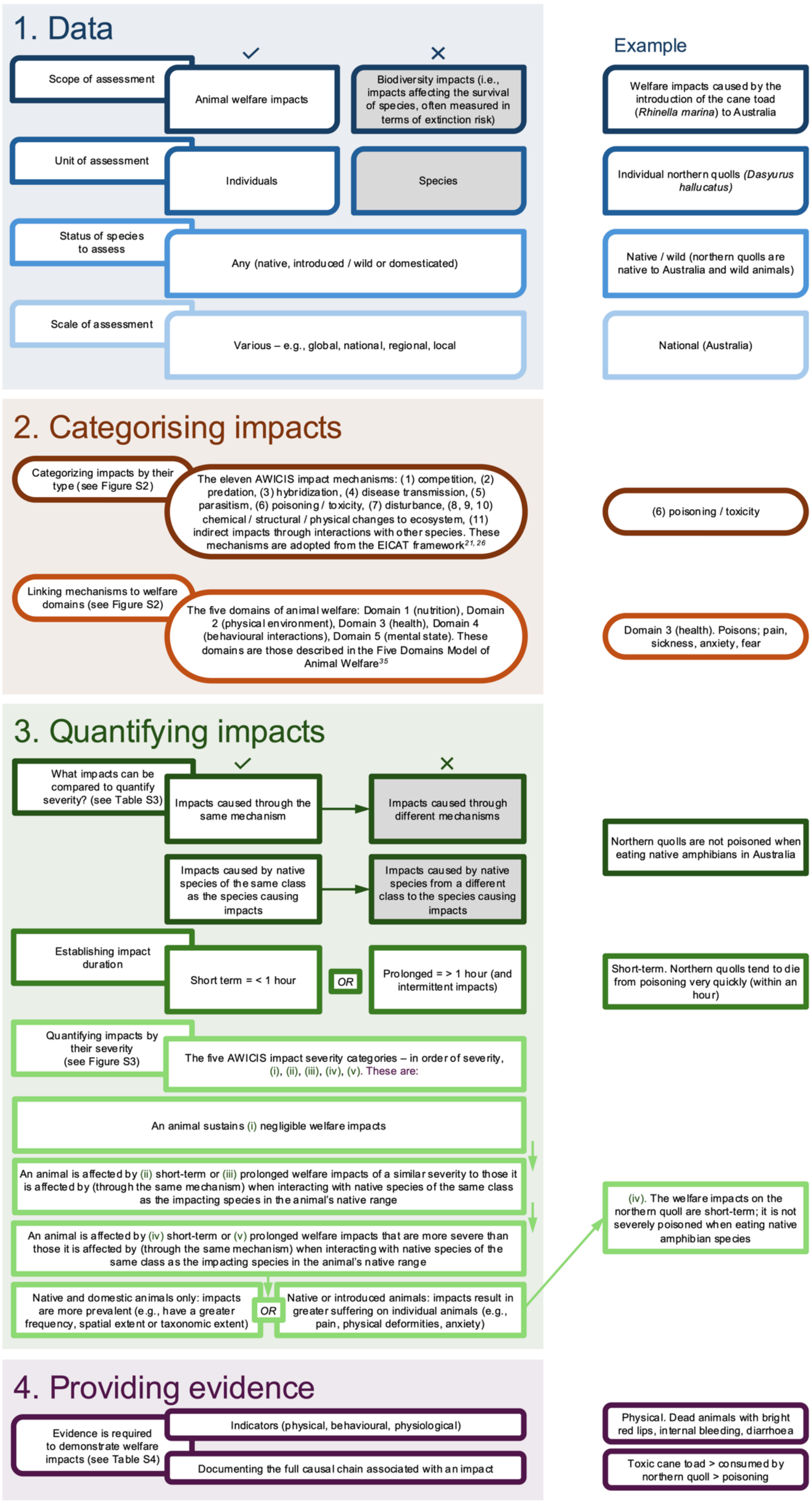

Figure S1. Key aspects of the AWICIS framework, including an impact example. This figure summarises four key aspects of AWICIS: (1) Data (what can be assessed under AWICIS), (2) Categorising impacts (the categories used to describe impacts under AWICIS), (3) Quantifying impacts (the scoring system used to assess the severity of impacts under AWICIS), (4) Providing evidence (the indicators that may be used to document impacts under AWICIS).

2. Categorising impacts

2.1 Impact mechanisms

AWICIS enables animal welfare impacts caused by biological invasions to be categorised by their type (*mechanism of impact*). It adopts 11 of the mechanisms identified for a related framework (the Environmental Impact Classification for Alien Taxa – EICAT) which assesses the impacts that introduced species have on biodiversity (e.g., competition, predation, hybridisation – for a complete list see IUCN, 2020, p. 11; Figure 1)[4]. Examples of interactions that cause adverse animal welfare impacts through these mechanisms are provided in Figure S2.

The EICAT mechanism 'grazing / herbivory / browsing' has not been included in the AWICIS framework, as this mechanism describes impacts to vegetation, and AWICIS considers welfare impacts on animals, not plants. Impacts associated with grazing of a poisonous / toxic plant species that cause animal suffering are included under the mechanism 'poisoning / toxicity'. Grazing that causes indirect welfare impacts by other species (e.g., grazing by herbivores that causes habitat loss, resulting in increased predation of an animal that can no longer find refuge) are included under the mechanism 'indirect impacts through interactions with other species'. The title of the EICAT mechanism 'biofouling or other direct physical disturbance' has been modified for AWICIS to 'disturbance', as biofouling (i.e., the inundation of physical structures with microorganisms, algae, bivalves etc.) is not considered to be key concern for animal welfare, but disturbance is.

Table S1. AWICIS – impact mechanisms.

Impact mechanism	Description of welfare impacts on an individual animal caused by interactions with other species (plants, fungi, animals etc.) as a result of biological invasions
(1) Competition	Competition for resources (e.g., food, refuge) between a species and an animal
(2) Predation	A species attacks / kills / eats an animal
(3) Hybridisation	Hybridisation between two animals
(4) Disease transmission	A species transmits a disease to an animal
(5) Parasitism	A species parasitises an animal
(6) Poisoning / Toxicity	A species poisons an animal
(7) Disturbance	A species disturbs an animal (e.g., through loud vocalisations, but not through other mechanisms identified in this table, such as competition or predation)
(8) Chemical changes to ecosystem	A species changes chemical characteristics of the environment (e.g., pH, nutrient levels) which results in welfare impacts on an animal (e.g., biological invasions can create anoxic conditions in river systems, leading to the suffocation of fish[9])
(9) Structural changes to ecosystem	A species changes the structure of the environment (e.g., species presence, abundance, diversity, complexity, spatial distribution, number of trophic levels) which results in welfare impacts on an animal (e.g., flammable and invasive introduced plants can change the structure of vegetation communities at the landscape scale, which become dominated by flammable plants, increasing the size of wildfires and the number of individual animals that they kill[10])
10) Physical changes to ecosystem	A species changes physical characteristics of the environment (e.g., thermal regimes, light regimes, water availability) which results in welfare impacts on an animal (e.g., introduced plants can alter thermal regimes of habitats, with lower and less variable temperatures reducing habitat suitability for native ectotherms[11])
(11) Impacts through interactions with other species	A species interacts with another species resulting in welfare impacts on another animal (e.g., introduced feral pigs (*Sus scrofa*) on Santa Cruz Island provided a food source for golden eagles (*Aquila* chrysaetos) which increased the eagle population size, and in turn the frequency of eagle predation on island foxes (*Urocyon littoralis*)[12])

The impact mechanisms described in this table are adopted (and amended) from a published framework developed to assess the impacts of introduced species on native biodiversity – the Environmental Impact Classification for Alien Taxa (EICAT)[1,4].

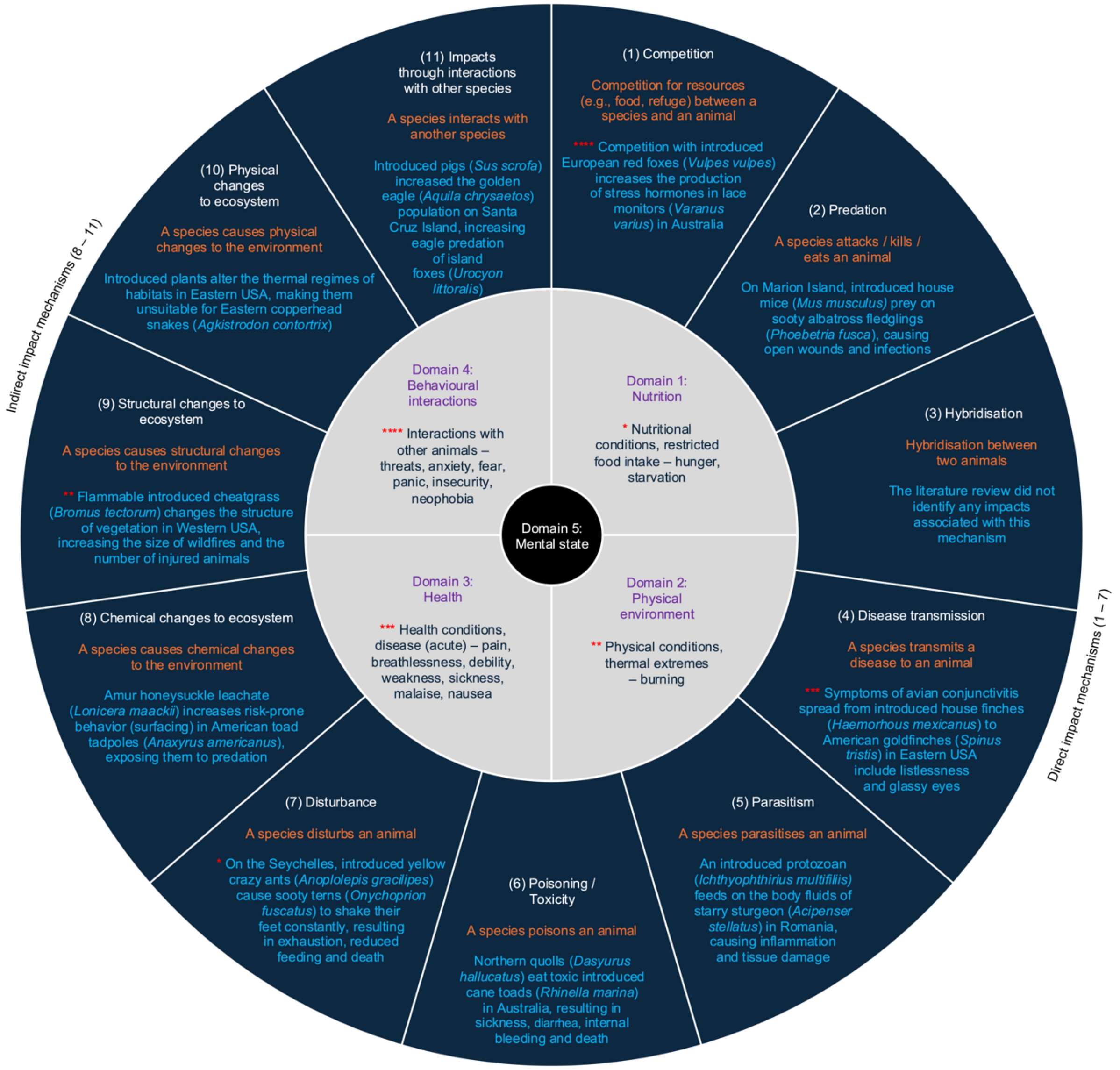

Figure S2. AWICIS – impact mechanism descriptions and examples. The impact mechanisms described in this figure are adopted (and amended) from a published framework developed to assess the environmental impacts of introduced species on native biodiversity – the Environmental Impact Classification for Alien Taxa (EICAT)[1]. These impact mechanisms have been amended to ensure their suitability to categorise animal welfare impacts (for details, see Section 2.1 of this document). White text = impact mechanism title; orange text = impact mechanism description; blue text = impact mechanism example. Impact mechanisms are linked to the '2020 Five Domains Model for Animal Welfare Assessment and Monitoring'[2] (inner circles, grey and black). Domain 4 has been amended to include animal-animal interactions (it previously only considered human-animal interactions). Domains 1 – 4 all contribute to effects on Domain 5 (the mental state of an animal), and hence so do the impact mechanisms. Four examples of linked domains and impact mechanisms are provided (red asterisks): * = Domain 1 (nutrition) / Mechanism 7 (disturbance); ** = Domain 2 (physical environment / Mechanism 9 (structural changes to ecosystem); *** = Domain 3 (health) / Mechanism 4 (disease transmission); **** = Domain 4 (behavioural interactions) / Mechanism 1 (competition). Example references: competition[13], predation[14], disease transmission[15], parasitism[16], poisoning / toxicity[7], disturbance[17], chemical changes to ecosystem[18], structural changes to ecosystem[19], physical changes to ecosystem[11], indirect impacts through interactions with other species[12].

2.2 Links with domains of animal welfare

AWICIS is linked to the *'2020 Five Domains Model for Animal Welfare Assessment and Monitoring'*[2] (Figure S2). Linking AWICIS with this model enables welfare impacts to be categorised at a finer scale than broad mechanism. The five domains are: (1) nutrition, (2) physical environment, (3) health, (4) behavioural interactions and (5) mental state. Domains 1 – 4 all affect Domain 5. Impacts caused by biological invasions involve interactions, and hence it might be considered appropriate to apply Domain 4 to all impacts. However, we use Domain 4 to categorise interactions that negatively affect an animal at the time of the interaction. Some interactions do not cause immediate negative effects; for example, transmission of a disease from one species to another, or parasitism of one species by another, does not necessarily involve negative, threatening or stressful interactions.

3. Quantifying impacts

3.1 Identifying relative changes to the welfare of an individual

Frameworks provide 'a way of organising things that can be easily communicated to allow for shared understanding, or that can be implemented to allow for generalisations useful for research, policy or management'[20]. As such, AWICIS aims to provide a simple method that can be used to quantify the animal welfare impacts of biological invasions by their severity. When assessing impacts on native animals, AWICIS does not attempt to distinguish between different levels of suffering associated with impacts caused by different mechanisms (e.g., the severity of welfare impacts caused by predation compared to those caused by a disease), or impacts caused through the same mechanism (e.g., predation), but by markedly different types of taxa that adopt different methods to cause these impacts (e.g., predation by a venomous snake vs predation by an ant vs predation by an owl). Nor does it attempt to identify and compare the severity of impacts affecting different types of animals, as the way they experience and are affected by impacts may differ[21]. Rather, AWICIS measures relative changes to the welfare of an individual animal caused by a specific impact mechanism associated with a biological invasion.

3.1.1 Impacts on native animals

To do so, the framework considers: (a) whether interactions caused by a biological invasion (through a specific mechanism, e.g., poisoning / toxicity) between a species (plant, animal, fungi etc.) and a native animal adversely affect the welfare of the native animal, (b) if so, whether these welfare impacts are more severe than those the native animal sustains when interacting with other native species of the same taxonomic class as the impacting species (and through the same mechanism), and (c) whether these impacts are short-term or cause prolonged suffering (see Section 3.2).

Thus, AWICIS identifies relative changes to the severity of the welfare impacts affecting an individual native animal at a given location. It acknowledges that all animals experience suffering[22] – it identifies unnecessary suffering caused by biological invasions. To do this AWICIS compares the severity of the impacts caused by biological invasions on a native animal, with the 'baseline' severity of the impacts that affect the native animal in the absence of biological invasions (i.e., those caused by other native species).

3.1.2 Impacts on introduced animals

As introduced animals may also suffer through interactions, AWICIS considers: (a) whether interactions caused by a biological invasion (through a specific mechanism) between a species (plant, animal, fungi etc.) and an introduced animal adversely affect the welfare of the introduced animal, (b) if so, whether these welfare impacts are more severe than those the introduced animal sustains in its native range when interacting with native species of the same taxonomic class as the impacting species (through the same mechanism), and (c) whether these impacts are short-term or cause prolonged suffering. Here, the severity of impacts sustained by the introduced animal as a result of its introduction to new regions are compared to the 'baseline' severity of the impacts that it sustains in its native range.

Introduced domesticated animals may not have an obvious native range, so it is not possible to make these comparisons. Hence for introduced domesticated animals, impacts they are affected by that are caused by a biological invasion are compared to impacts caused by native species within the country where the impact occurs.

3.1.3 Prevalence

For native animals, AWICIS also considers whether the impacts caused by a biological invasion (through a specific mechanism) increase the prevalence of existing impacts caused by native species in the absence of the biological invasion. Such increases may arise because the spatial extent or taxonomic extent of these impacts are increased, or because their frequency is increased.

As the impacts of biological invasions on introduced wild animals do not occur in their native range, changes to the prevalence of the impacts they are affected by would be difficult to assess. However, for introduced domesticated animals, as impacts caused by biological invasions that affect them are compared to impacts caused by native species within the country where the impact occurs, prevalence can be assessed.

3.2 Short-term vs prolonged impacts

From an animal welfare perspective, impacts that do not cause prolonged suffering are considered to be less harmful. To distinguish between short-term and prolonged impacts, AWICIS stipulates an approximate duration of < 1 hour for short-term impacts, with any impact lasting longer than 1 hour being categorised as prolonged. In terms of affective state, this equates to a rough distinction between short-term emotional responses to a specific event, and the onset of longer-term affective states including moods and, eventually, more prolonged affective disorders[23]. Intermittent impacts that occur over long periods (> 1 hour) but not constantly (e.g., regular but intermittent competitive interactions with an aggressive animal over the course of a breeding season) should be categorised as prolonged.

3.3 Taxonomic extent of assessments

The characteristics of welfare impacts caused through a specific mechanism (e.g., predation) may vary depending on the taxon causing the impacts. For example, predation by a fire ant involves different processes to predation by a venomous snake or predation by a bird of prey. As such, it may be difficult to compare the severity of the welfare impacts caused through the same mechanism but by different taxa. AWICIS therefore stipulates that comparisons of the severity of the welfare impacts caused by biological invasions through a specific mechanism should be made amongst species of the same class (e.g., predation of an animal by a bird resulting from a biological invasion should be compared with predation of that affected animal by other birds in its native range). This enables mechanisms that involve broadly the same types of processes to be compared in terms of the severity of their welfare impacts. Nevertheless, this not an exact method – there will be cases where species from the same class do not cause impacts through the same mechanism using the same processes (e.g., predation by a constricting snake vs a venomous snake). The assessor must use their judgement to identify such cases in order to avoid making inappropriate comparisons to establish impact severity.

3.4 Impact severity categories

Taking into account the preceding information in Section 3, AWICIS uses five impact categories to quantify the severity of animal welfare impacts arising from biological invasions (Figure S3; Table S2).

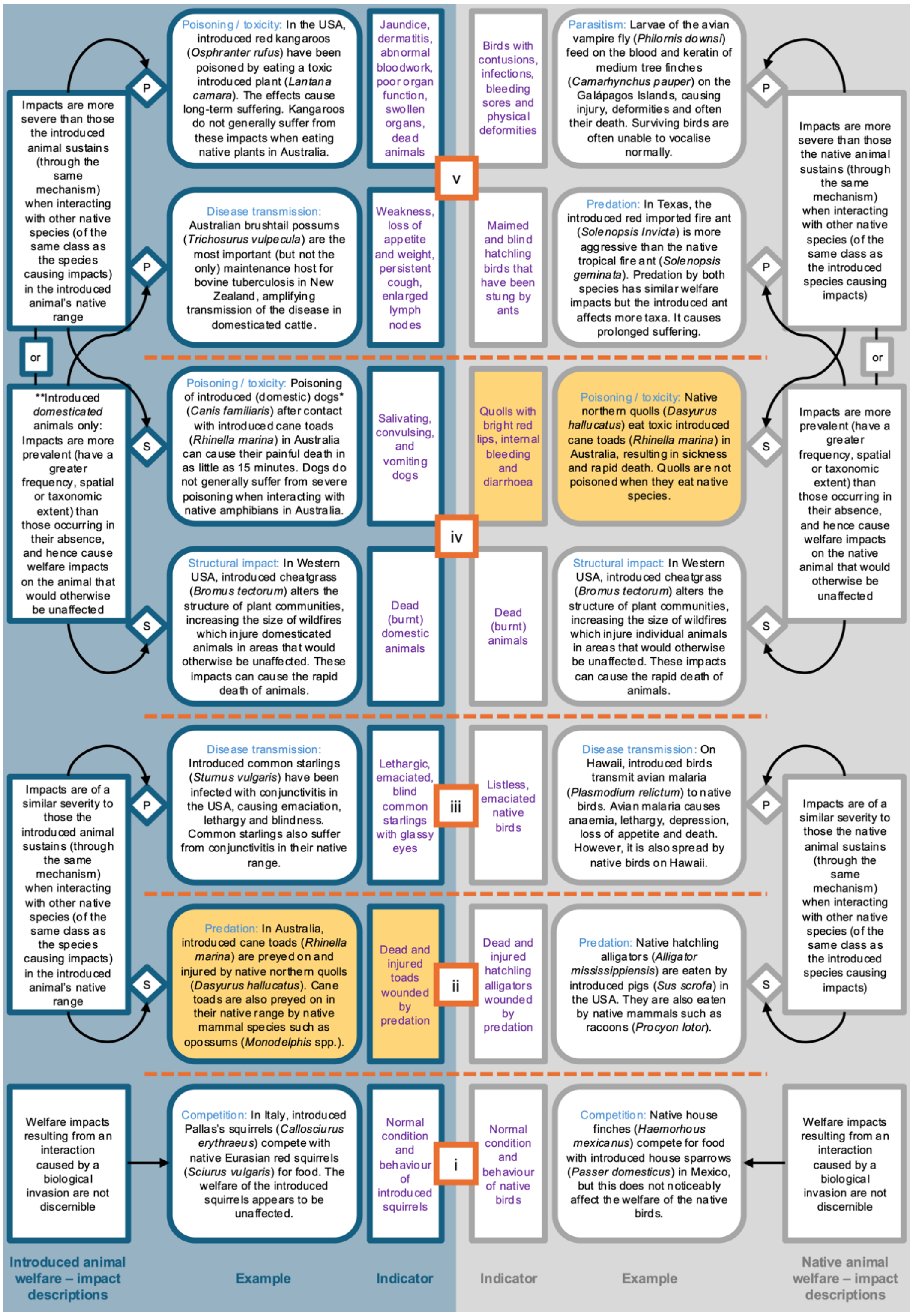

Figure S3. AWICIS – the five impact severity categories. Red squares = impact severity categories, in increasing order of severity: (i), (ii), (iii), (iv), (v). P = prolonged impact; S = short-term impact. Light blue text = impact mechanism associated with each example. Purple text = indicator of welfare impacts. Light orange boxes = a linked example that affects both interacting species. References – introduced animal welfare examples: (i) – competition[24], (ii) – predation[7], (iii) – disease transmission[25], (iv) – structural impact[19], (iv) – poisoning / toxicity[26], (v) – disease transmission[27], (v) – poisoning / toxicity[28]. References – native animal welfare examples: (i) – competition[29], (ii) – predation[30], (iii) – disease transmission[31], (iv) – structural impact[19], (iv) – poisoning / toxicity[7], (v) – predation[32–35], (v) – parasitism[36]. *As introduced domesticated animals do not have an obvious native range to compare impacts to, impacts affecting them that are caused by a biological invasion are compared to impacts caused by native species within the country where the impact occurs. **Hence, prevalence can be assessed for affected introduced domesticated animals (in the same manner as for affected native animals). However, for introduced wild animals, as the impacts caused by the biological invasion do not occur in the introduced animal's native range, changes to prevalence cannot be assessed.

Table S2. AWICIS – impact severity categories.

<table>
<tr><th>Description</th><th>Duration (see Section 3.2)</th><th>Impact category</th></tr>
<tr><td>An animal (native or introduced, wild or domesticated) sustains negligible welfare impacts.</td><td>Any</td><td>(i)</td></tr>
<tr><td rowspan="2">An animal is affected by welfare impacts (through a specific mechanism) that are of a similar severity to those it is affected by (through the same mechanism) when interacting with native species of the same class as the impacting species in the animal's native range.</td><td>Short-term</td><td>(ii)</td></tr>
<tr><td>Prolonged</td><td>(iii)</td></tr>
<tr><td rowspan="2">An animal is affected by welfare impacts (through a specific mechanism) that are more severe than those it is affected by (through the same mechanism) when interacting with native species of the same class as the impacting species in the animal's native range.

For native and introduced animals, this may be because the impacts caused by a biological invasion result in greater suffering than those occurring in their absence (e.g., pain, anxiety, problems associated with physical deformities) (see Table S3).

Additionally, for native animals only, this may be because the impacts are more prevalent than those occurring in their absence (e.g., they have a greater frequency, spatial extent or taxonomic extent) and hence cause impacts on an individual animal that would otherwise be unaffected. This scenario does not apply to introduced animals, as the impacts caused by biological invasions do not occur in the introduced animal's native range, and hence prevalence cannot be assessed (see Table S3). However, as introduced domesticated animals do not have an obvious native range to compare impacts to, impacts affecting them caused by a biological invasion are compared to those caused by native species within the country where the impact occurs. Hence, prevalence can be assessed for affected introduced domesticated animals (in the same manner as for affected native animals).</td><td>Short-term</td><td>(iv)</td></tr>
<tr><td>Prolonged</td><td>(v)</td></tr>
</table>

3.5 Establishing impact severity

Impacts are assigned to any affected animal. To establish the appropriate impact severity score, the severity of the welfare impacts it is affected by that are caused by a biological invasion are compared with those that it is affected by in the absence of biological invasions (see Table S3 for examples of how to determine category (iv) and (v) impacts).

Impacts on animals in their native ranges that occur in the absence of biological invasions may rarely be studied and reported (impacts caused by native species on other native species are less likely to attract research when compared to impacts caused by biological invasions which pose a threat to native biodiversity). Therefore, in order to prevent issues associated with a lack of data from which to compare and assess impacts, AWICIS stipulates that impacts occurring in the absence of biological invasions do not have to be published (although published data can be used if available) – rather, they can be inferred based on the native species present in a region and their likely interactions. These inferences can be made by reviewing the life-history traits (e.g., diet and habitat preferences) of the native species – a process that has been successfully used to identify likely interactions involving native birds and predatory introduced birds[37]. As AWICIS stipulates that comparisons must be made between welfare impacts caused by the same mechanism and by the same class of animal (e.g., predation of an animal by a bird resulting from a biological invasion should be compared with predation of that affected animal by other native birds in its native range) this is a focused task (i.e., it does not require consideration of the impacts caused by all native species in a region).

Table S3. AWICIS – identifying category (iv) and (v) impacts.

**Category (iv) or (v) impact description**		**Status of affected animal**	**Example measures to assess impact severity**	**Examples**	**Impact severity**
An animal is affected by (iv) short-term or (v) prolonged welfare impacts (through a specific mechanism) that are more severe than those it is affected by (through the same mechanism) when interacting with native species of the same class as the impacting species in the animal's native range.	The impacts caused by a biological invasion result in greater suffering than those caused in their absence in the affected animal's native range (e.g., pain, problems associated with physical deformities, anxiety).	Native or introduced.	Impact duration. Does an animal suffer from welfare impacts arising from biological invasions that are more protracted than those it suffers from (through the same mechanism) when interacting with native species (of the same class as the impacting species) in its native range?	Slow, protracted predation of native house sparrows (*Passer domesticus*) by introduced domestic cats (*Felis catus*)[38], compared to efficient predation of house sparrows by native mammals such as weasels (*Mustela nivalis*).	(iv) if the impacts are short-term, or (v) if they are prolonged.
				Inefficient predation of European rabbits (*Oryctolagus cuniculus*) by introduced domestic dogs (*Canis familiaris*) in the UK, which causes additional anxiety, stress and pain through long chases and protracted kills[39] when compared to efficient predation of European rabbits by native mammals such as red foxes (*Vulpes vulpes*).	
			Pain / injury / physical condition. Does an animal suffer from welfare impacts arising from biological invasions that are more painful / injurious / damaging than those it is affected by (through the same mechanism) when interacting with native species (of the same class as the impacting species) in its native range?	Native finch species (*Geospiza* spp.) on the Galápagos suffering from injuries and physical deformities as a result of parasitism by the introduced avian vampire fly (*Philornis downsi*)[36]. Native finch species do not suffer from physical deformities as a result of parasitism by native insects on the Galápagos.	
				Aggressive introduced common mynas (*Acridotheres tristis*) attacking and injuring native echo parakeets (*Psittacula eques*) when competing for resources in Mauritius[40]. Echo parakeets compete for resources with other native bird species in Mauritius without damaging physical interactions.	
			Abnormal behaviour. Does an animal suffer from welfare impacts arising from biological invasions that cause it to behave in a manner not observed when interacting with native species (of the same class as the impacting species) through the same mechanism in its native range?	Competition for nest-cavities with aggressive introduced rose-ringed parakeets (*Psittacula krameri*) in Belgium causes native Eurasian nuthatches (*Sitta europaea*) to abandon their nests[41]. They do not abandon their nests when competing with native birds in Belgium.	
				Repetitive foot shaking by native sooty terns (*Onychoprion fuscatus*) that are being disturbed by ants in Seychelles[42]. Sooty terns do not behave in this manner in the absence of introduced ant invasions in Seychelles.	
	The impacts caused by a biological invasion are more prevalent than those occurring in their absence (e.g., they	Native only (does not apply to introduced animals, as the impacts they are affected by do not occur in the introduced	Frequency. Does the biological invasion increase the frequency of animal welfare impacts, such that an individual animal suffers from welfare impacts when it otherwise wouldn't?	Some introduced species are considered to be amplifiers of local pathogens[43]. For example, Australian brushtail possums (*Trichosurus vulpecula*) are the most important (but not the only) maintenance host for bovine tuberculosis in New Zealand, providing interface for transmission between livestock and wild forest animals[27].	(iv) if the impacts are short-term, or (v) if they are prolonged.
			Spatial extent. Does the biological invasion increase the spatial extent of animal welfare impacts, such that an individual	Flammable introduced cheatgrass (*Bromus tectorum*) alters the structure of plant communities across landscapes, increasing the size of wildfires which injure and kill individual animals in regions that were unaffected by fires prior to the biological invasion (e.g., greater sage grouse (*Centrocercus urophasianus*) in the Northern Great Basin of Western USA[10]).	

Category (iv) or (v) impact description		Status of affected animal	Example measures to assess impact severity	Examples	Impact severity
	have a greater frequency, spatial extent or taxonomic extent) and hence cause impacts on individual native animals that would otherwise be unaffected.	animal's native range, and hence prevalence cannot be assessed). However, as introduced domesticated animals do not have an obvious native range to compare impacts to, impacts affecting them caused by a biological invasion are compared to those caused by native species within the country where the impact occurs. Hence, prevalence can be assessed for affected introduced domesticated animals (in the same manner as for affected native animals).	animal suffers from welfare impacts in a region where it otherwise wouldn't?	In Florida, the introduced red imported fire ant (*Solenopsis Invicta*) is more aggressive than the native tropical fire ant (*Solenopsis geminata*)[32]. Predation by both species may have similar welfare impacts[33], but the impacts caused by the introduced species are more widespread. For example, a survey of turtle nests on Florida beaches found red imported fire ants at 431 nests and the tropical fire ant at just three[35]. Information used to establish the severity of the impacts caused by the introduced species includes the spatial extent of the impacts caused by the native species (inferred and / or reported).	
			Taxonomic extent. Does the biological invasion increase the breadth of taxa that suffer from welfare impacts, such that an individual animal suffers from welfare impacts when it otherwise wouldn't?	In the USA, the introduced red imported fire ant (*Solenopsis Invicta*) is more aggressive than the native tropical fire ant (*Solenopsis geminata*)[32]. Predation by both species may have similar welfare impacts[33], but the introduced ant causes the suffering of a greater variety of animals[44,45]. Information used to establish the severity of the impacts caused by the introduced species includes the taxonomic extent of the impacts caused by the native species (inferred and / or reported).	

3.6 Assessing diverse impacts on an individual animal

An animal may be assigned different impact scores associated with the welfare impacts it is affected by that occur through different mechanisms. Hypothetical impact severity scores for adverse animal welfare impacts on a bird introduced to an island could include: a disease transmitted by a related bird species on the island that the introduced bird does not suffer from in its native range, the impacts are prolonged – impact severity score = (v); competition with other birds for food, similar to that experienced in the introduced bird's native range, the impacts are prolonged – impact severity score = (iii); predation of the introduced bird by a mammal on the island, but mammals also prey on the bird in its native range, the impacts are short-term – impact severity score = (ii).

3.7 Comparing impact severity across affected individuals

Impacts affecting different individual animals through the same mechanism and assigned the same severity score (e.g., (iv)) may actually be more or less severe than one another depending on the different process associated with that mechanism. However, AWICIS does not directly compare the severity of welfare impacts affecting different individual animals. Rather, it identifies and quantifies relative changes to the severity of the welfare impacts experienced by an individual animal through a specific mechanism that occur as a result of a biological invasion. Hence, two animals with impacts scores of (iv) (for example) may suffer from different types of impacts that can't be compared, but these two animals have in common a shared characteristic – they are both affected by short-term welfare impacts that are more severe than those they experience in the absence of the biological invasion.

3.8 Consideration of welfare impacts that are not caused by biological invasions

Existing impacts on animals not caused by biological invasions may cause severe, prolonged suffering – greater than the suffering caused by biological invasions (e.g., predation caused by a biological invasion, scored as (ii), compared to a parasite prevalent in the native species assemblage that causes long-term suffering of animals). Nevertheless, AWICIS does not score the severity of existing welfare impacts in native communities – it aims to identify and quantify changes to the severity of welfare impacts caused by biological invasions.

3.9 Numbers of affected individuals

Some risk assessment methods for farmed animals consider the sheer number of individuals that are affected by a welfare issue as an indicator of its severity (e.g., the number of individual pigs that suffer from tail biting)[46]. However, abundance data for many introduced species is unavailable, and hence establishing the scale of their impacts in terms of affected individual animals would be difficult – this is why abundance was not used as a variable in a study that identified factors that cause introduced birds

to have severe biodiversity impacts[47]. Furthermore, frameworks should provide simple methodologies that enable trends in data to be easily communicated[20] – whilst it may be relatively straightforward to estimate numbers of affected animals within a 'closed system' such as a farm, it is often difficult to calculate with confidence the sheer number of individual animals that may be affected by a biological invasion. For these reasons, AWICIS avoids using numbers of affected individuals as a measure of impact severity. Nevertheless, AWICIS assessments may inform additional research that estimates the number of animals that are affected by a biological invasion (i.e., by providing information on the severity and type of welfare impacts assessed using AWICIS, which may be combined with information on the sheer number of affected animals).

4. Evidence of impacts

4.1 Impacts occurring under natural conditions

AWICIS assessments should only be undertaken using evidence of impacts collected under natural conditions. Evidence of impacts established through laboratory experiments should not be used. Impacts occurring in the wild but that are mediated by human actions can be included – hence impacts affecting the welfare of domesticated animals can be assessed.

4.2 Physical, behavioural and physiological indicators

The welfare of an animal may be assessed using observations and measures of behavioural, physiological and physical indicators (Table S4). Hence when assessing welfare impacts using AWICIS, specific indicators must be documented[48]. Validation of these indicators as accurate markers of an animal's affective state is a key area of animal welfare science. A framework for achieving validation is set out in Mason *et al.* (in press)[49], and includes tests such as whether the indicator changes reliably in animals exposed to preferred or avoided stimuli, or to those associated with fitness benefits and harms. It is possible, even likely, that such validation will not have been achieved in a wild species of interest. In such cases, it is desirable that there has been validation in related (even domesticated) species or taxa and/or that the indicator is widely recognised as a useful marker of a positive or negative state (e.g. tissue damage or disease and associated sickness behaviour).

A commonly used physiological indicator is glucocorticoid level ('stress hormone': cortisol, corticosterone) measured from blood, urine or faecal samples – this has been used to identify stress in wild animals caused by biological invasions (see Table S4). Whilst this may provide useful information concerning an organism's arousal state, which could be linked to its response to an aversive welfare challenge, on its own it does not provide definitive evidence as to whether the animal is in a negatively (e.g. fear-like) or positively (e.g. excitement) valenced affective state. Because these are widely used markers in studies of wild animal stress and welfare, we do refer to them here (e.g. Figure S2; Figure

S3) but acknowledge that they are most useful when accompanied by other measures of affective valence.

Table S4. AWICIS – indicators of animal welfare impacts.

Indicator	Definition	Examples
Physical	The physical appearance of an animal which can be observed visually (e.g., injury, disease, body condition[50]). For example, an arched back can be used to detect pain in some animals[51].	White spots on fish in the Danube River affected by the introduced parasite *Ichthyophthirius multifiliis* which causes irritation, inflammation, and reduced growth and immune function[16]
		Poor physical condition (e.g., weight loss) of Eurasian red squirrels (*Sciurus vulgaris*) competing for food with introduced Pallas's squirrels (*Callosciurus erythraeus*) in Italy[24]
		American goldfinches (*Spinus tristis*) with glassy eyes caused by conjunctivitis spread by introduced house finches (*Haemorhous mexicanus*) in the USA[15]
Behavioural	The behaviour of an animal which can be observed directly or via recorded observations (e.g., avoidance, abnormal behaviour, restriction of activity, posture, facial expressions, vocalisations). For example, repetitive behaviour in animals has been used as an indicator of stress and boredom[51].	Livestock excessively salivating after eating a toxic introduced plant (*Parthenium hysterophorus*)[52]
		Darwin's finches (*Geospiza* spp.) that can't vocalise (deformed beaks) due to parasitism by the avian vampire fly (*Philornis downsi*) on the Galápagos[36]
		Repetitive shaking of feet by sooty turns (*Onychoprion fuscatus*) disturbed by yellow crazy ants (*Anoplolepis gracilipes*) on the Seychelles[42]
		Lethargic albatrosses (*Phoebetria fusca*) dying from infections and injuries caused by predation by introduced house mice (*Mus musculus*) on Marion Island[14]
Physiological	Measurable biological functions that provide insights into the welfare of an animal (e.g., heart rate, stress hormone levels)[53].	Elevated levels of glucocorticoid metabolites (a stress hormone) in faecal samples of native Eurasian red squirrels (*Sciurus vulgaris*) resulting from aggressive interactions with introduced grey squirrels (*Scurius carolinensis*) in Italy[54]
		Lower levels of sex steroid metabolites and elevated levels of corticosterone metabolites (a stress hormone) in urine samples of native Fijian ground frogs (*Platymantis vitiana*) due to competition with introduced cane toads (*Rhinella marina*) on Viwa Island[55]
		Elevated levels of corticosterone (a stress hormone) in native monitor lizards (*Varanus varius*) in Australia, caused by competition with European red foxes (*Vulpes vulpes*)[13]

4.3 Describing the full causal chain associated with an impact

Some welfare impacts are direct, such as predation of one animal by another – the processes associated with direct impacts tend to be relatively intuitive and straightforward to document. However, other welfare impacts caused by biological invasions are indirect (e.g., changes to the structure of vegetation communities at the landscape scale, caused by the introduction of cheatgrass (*Bromus tectorum*) to Western USA – because cheatgrass is flammable it increases the size of wildfires, and in turn the number of individual animals that they injure and kill)[19]. In such cases, it is important to describe the full causal chain leading to impact, in order to clearly demonstrate how welfare impacts on animals arise as a result of a biological invasion. The AWICIS assessment template (Section 7 of these guidelines) includes the requirement to describe the causal chain associated with welfare impacts.

5. Assessment scale

A comprehensive assessment would compare the severity of welfare impacts affecting an animal caused by a biological invasion with those that are caused by native species across the animal's entire native range in the absence of the biological invasion. However, as with EICAT, assessments may be undertaken at varying scales (local, regional, national, global). Local assessments would be those associated with a specific location (e.g., a city or an island); regional assessments would be those associated with a wider region, including island groups (but not a country); national and global assessments would be for a specific country or for the world, respectively. For example, it may be more informative to compare the severity and type of the welfare impacts caused by a biological invasion on an individual animal at the local scale (e.g., a specific region with a distinct native species assemblage such as an urban area) with the severity of the impacts it would sustain in the absence of the biological invasion in that region (rather than across the animal's entire native range). This would inform management strategies to improve animal welfare for that region. Assessments undertaken at the national scale would inform national management strategies to address the welfare impacts of biological invasions. Nevertheless, and as with EICAT, varying scales of assessment may lead to different impact severity scores for an affected animal – welfare impacts may be more severe for assessments undertaken at the local scale, where there are likely to be fewer native species to compare impacts with (and hence the welfare impacts of biological invasions are likely to be more severe), in comparison to national assessments.

For an introduced animal, it is also possible to undertake assessments as different spatial scales, for example, to compare the impacts it is affected by as a native animal in only a specific part of its native range (e.g., a country) to determine whether the impacts in that country are less harmful than those it is affected by as an introduced animal. Nevertheless, comparative assessments of welfare impacts on different animals should be undertaken at the same scale in order to facilitate meaningful comparisons regarding the severity of these impacts.

For domesticated (introduced) animals (that do not have an obvious native range from which to establish impact severity), assessments should be completed at the country scale, and within the country where the impact occurs (i.e., impacts would be compared to those sustained by the domesticated animal that are caused by native species in the country of impact). Hence, if comparisons of the severity of welfare impacts on wild vs domesticated animals are required, assessments on wild animals should also be completed at this scale.

6. Assessment confidence

Many factors may affect the assessor's confidence that the impact severity category they have allocated to an impact is correct, including for example: the quality of the published information describing an

impact; uncertainty regarding the spatial distribution of an animal, and hence its likelihood of being affected by an impact; uncertainty regarding the relative severity of an impact caused by a biological invasion when compared to existing impacts caused in its absence; uncertainty regarding the status (native or introduced) of the organism causing an impact; uncertainty regarding the duration of an impact (short-term or prolonged). To account for this, AWICIS includes three confidence categories (in a similar manner to EICAT) to report the level of confidence an assessor has that the impact severity category they have assigned to an impact is correct: 'low', 'medium' or 'high'. A brief written explanation to justify why a specific confidence category has been chosen is also required for each assessment. Although subjective, these categories help to identify possible discrepancies and knowledge gaps regarding the animal welfare impacts of biological invasions. They also enable data of varying quality to be reported and assessed, whilst clearly acknowledging and documenting its shortcomings.

7. AWICIS assessment template

Assessments undertaken using AWICIS should be completed using the assessment template which has been uploaded to an online repository[56]. Filling out the template will help to ensure that an assessment considers all aspects of the AWICIS assessment process. The template includes several impact assessment examples.

Supplementary Note 2

Testing the framework

Methods

We adopted a three-stage process to test the framework. First, we asked three research scientists with no prior knowledge of AWICIS to independently review the draft guidelines and assessment template and provide comments. These comments resulted in some minor amendments to the guidelines and assessment template. For example, Assessor 3 noticed some inconsistencies in the way assessments were described in the guidelines in comparison to the template, and Assessor 2 noted that although the guidelines included instructions on how to assess impacts affecting introduced domesticated animals, these instructions needed to be included in the legend to Figure S3 in Supplementary Note 1.

Second, we asked the same three research scientists to remotely undertake a 'training' exercise, whereby they used AWICIS to assess five impact examples. To standardise the assessment process (so that we could directly compare results across the three assessors) we provided each assessor with the AWICIS assessment template with the following information for each of the five impact examples:

- the common and scientific name of the welfare affected animal.
- the common and scientific name of the interacting species.
- the reference article to be reviewed in order to assess the impact.

The assessors were asked to complete all remaining sections of the reporting template for each of the five impact examples. This initial training exercise enabled the assessors to familiarise themselves with the AWICIS reporting template and the assessment process. All three assessors provided feedback which helped to improve the instructions provided in the assessment template. For example, based on the feedback provided, we added instructions to the template to enter *'NA'* for the assessment of impact prevalence if the welfare affected animal was introduced (Row 2, Column AL of the assessment template).

Third, we provided the assessors with a further ten impact examples (with the same information as described above), which they were asked to assess without any advice. We made sure that the version of the guidelines reviewed by the assessors did not include information on these ten impact examples. Adopting the assessment process used for formal EICAT assessments undertaken by the IUCN EICAT Authority, these AWICIS assessments were then reviewed by T.E. and returned to the assessors to discuss any issues (e.g., misunderstandings or oversights – examples are provided in the 'Results' section below). The reviewers agreed with all review comments (i.e., following review, there were no major differences of opinion regarding the assessment process and outcomes).

Results

The three assessment templates with the examples completed by the three assessors have been uploaded to a public repository[57]. For the final ten examples, the templates include the assessors first assessment attempts (i.e., not the reviewed and updated assessments) – hence they include errors made by assessors and comments provided by T.E. All comments in the templates have been anonymised. A results summary for three aspects of the final ten assessments (impact severity, impact mechanism and confidence rating) is provided in Table S5 below. These results are of the first assessment attempts (not the reviewed and updated assessments). With regard to impact severity, of the 30 examples assessed (3 x 10 impact examples), 26 (87%) were allocated to the expected impact severity category (shaded green in Table S5). Errors can be attributed to the assessors learning how to use an unfamiliar framework (rather than due to fundamental flaws with the framework) – for example, Assessor 1 assessed an impact as being short-term, but then incorrectly allocated the impact to an impact category associated with prolonged impacts (level v) (Assessment template 1 – Row 12, Column AM). When prompted with a comment that the impact severity category needed checking, the assessor allocated it to the correct impact category (level iv). Assessor 1 also assessed a welfare impact on Roe deer (*Capreolus capreolus*) caused by competition with Crested porcupines (*Hystrix cris*) as being level (ii), when actually the impact was more severe – after review, the assessor acknowledged that they had assessed the impact of competition between the two animals at feeding stations, but had forgotten to consider the injuries to deer that were caused by porcupine quills (Assessment template 1 – Row 9, Column AM).

With regard to impact mechanisms, 25 of the 30 assessments (83%) were categorised correctly (shaded green in Table S5). Again, the errors were due to inexperience in use of the framework – for example Assessor 3 categorised an impact caused by a parasitic flea as being 'Disease transmission' when it could more accurately have been categorised as 'Parasitism' (Assessment template 3 – Row 13, Column Z). Two assessors incorrectly categorised an 'Indirect impact through interactions with other species' as being 'Predation' (Assessment templates 1 and 3 – Row 12, Column Z). After discussion with one of the assessors, it became apparent that the way the impact example had been structured in the template by T.E. had caused some confusion. The impact was for increased predation of Macquarie Island parakeets (*Cyanoramphus novaezelandiae erythrotis*) on Macquarie Island – this increase was caused by the introduction of European rabbits (*Oryctolagus Cuniculus*), which provided food for feral cats (*Felis catus*), increasing the cat population on the island and hence the prevalence of cat predation of individual birds. T.E. had included the feral cat in the template as the first interacting species, and the European rabbit as the second interacting species, when actually the European rabbit should have been included as the first interacting species (as this is the species causing the indirect impact) and the feral cat should have been included as the second interacting species (as increased predation by feral cats was the outcome – the secondary mechanism). We have updated the instructions in the assessment template to provide advice on the order in which interacting species should be listed for

the mechanism 'Impacts caused by interactions with other species' (see Row 1, Columns L – O of the assessment template). The assessors confirmed that this would improve the assessment process for this mechanism by making it more intuitive.

With regard to confidence levels, although they varied across assessor, there was some consistency as no impact that was assessed as 'low' confidence by one assessor was assessed as 'high' confidence by another or vice versa (Table S5). Impact assessments were either assessed as being of 'high' confidence by all assessors; 'high' confidence by some assessors and 'medium' confidence by others; or 'medium' confidence by some assessors and 'low' confidence by others. Variation here could be explained by differences in the personality of the assessors, as some assessors may be more cautious than others; similarly, this variation may also be explained by differences in knowledge and experience.

Table S5. Results of ten assessments carried out by three assessors to test the AWICIS framework and assessment template.

	Assessor 1	Assessor 2	Assessor 3
Impact severity	v	v	v
	ii	ii	ii
	v	v	v
	v	v	v
	ii	v	v
	ii	i	i
	iii	v	v
	v	iv	iv
	i	i	i
	v	v	v
Impact mechanism	Parasitism	Parasitism	parasitism
	Predation	Predation	Predation
	Poisoning / toxicity	Poisoning / toxicity	Poisoning / toxicity
	Chemical impact on ecosystem	Physical changes to ecosystem	Chemical impact on ecosystem
	Competition	Competition	Competition
	Competition	Competition	Disturbance
	Disease transmission	Disease transmission	Disease transmission
	Predation	Interactions with other species	Predation
	Parasitism	Competition*	Disease transmission
	Competition	Competition	Competition
Confidence level	High	High	High
	Medium	Medium	Low
	Medium	Medium	Low
	Medium	Medium	Low
	Medium	High	High
	High	Medium	High
	Medium	High	Medium
	High	Medium	High
	Low	Medium	Low
	High	Medium	Medium

Impact severity = the impact severity score assigned by an assessor to each impact example (green = expected impact score; unshaded = unexpected impact score). Impact mechanism = the type of impact assigned by an assessor to each impact example (green = expected mechanism; unshaded = unexpected impact mechanism). Confidence level = the level of confidence the assessor had regarding the likelihood of their assessment being correct. Confidence categories are shaded for visual reference: 'high' (red); 'medium' (orange); 'low' (yellow). * = the assessor considered the effects of 'competition' for this assessment which is correct as the two squirrel species in the example do compete for resources. However, one species transmits a parasitic flea to the other, so 'parasitism' could also have been assessed here (as done by Assessor 1 and Assessor 3 – although Assessor 3 incorrectly categorised the impact mechanism as being 'disease transmission').

Supplementary Note 3

Contingency table tests (unconditional exact tests calculated using the FunChisq package)[58].

(i) Bird invasions

Table S6. Contingency table (unconditional exact test) showing the number of animal welfare impacts associated with bird invasions, as distributed by their severity and order of affected animal.

	Passeriformes (perching birds)	Psittaciformes (parrots)	Charadriiformes (shorebirds and allies)	Procellariiformes (seabirds)	Other bird orders	Marginal row totals
Less severe	359	92	24	12	279	766
More severe	25	5	22	14	11	77
Marginal column totals	384	97	46	26	290	843

Statistic = 57.8, parameter = 4, $P < 0.001$, sample estimates: non-constant unconditional function index = 0.17. Due to small sample sizes, data for two classes (mammals and reptiles) were removed for this test (mammals = 3 'less severe' impacts and 2 'more severe' impacts; reptiles = 8 'less severe' impacts and 0 'more severe' impacts). Hence the test only analysed data for birds. Also due to small sample sizes, several bird orders were merged to form the 'Other bird orders' category: Accipitriformes (diurnal birds of prey), Anseriformes (waterfowl), Bucerotiformes (hornbills and hoopoes), Caprimulgiformes (nightjars and allies), Coliiformes (mousebirds), Columbiformes (pigeons and doves), Coraciiformes (kingfishers and allies), Cuculiformes (cuckoos, hoatzins and turacos), Falconiformes (falcons and caracaras), Galliformes (game birds), Gaviiformes (loons), Gruiformes (cranes, rails and allies), Pelecaniformes (pelicans, herons, ibises and allies), Phaethontiformes (tropicbirds), Phoenicopteriformes (flamingos), Sphenisciformes (penguins), Strigiformes (owls) and Suliformes (gannets, cormorants and allies).

Table S7. Contingency table (unconditional exact test) showing the number of animal welfare impacts associated with bird invasions, as distributed by their severity and mechanism.

	Competition	Predation	Other mechanisms	Marginal row totals
Less severe	509	153	115	777
More severe	9	65	5	79
Marginal column totals	518	218	120	856

Statistic = 148.8, parameter = 2, $P < 0.001$, sample estimates: non-constant unconditional function index = 0.34. Due to small sample sizes, three mechanisms were merged to form the 'Other mechanisms' category: Hybridisation, Disease transmission and Parasitism (brood).

Table S8. Contingency table (unconditional exact test) showing the number of animal welfare impacts associated with bird invasions, as distributed by their severity and location of impact (island or other location).

	Island	Other location	Marginal row totals
Less severe	211	566	777
More severe	72	7	79
Marginal column totals	283	573	856

Statistic = 117.4, parameter = 1, $P < 0.001$, sample estimates: non-constant unconditional function index = 0.37.

(ii) Ant invasions

Table S9. Contingency table (unconditional exact test) showing the number of animal welfare impacts associated with ant invasions, as distributed by their severity and class of affected animal.

	Birds	Other classes	Marginal row totals
Less severe	5	4	9
More severe	47	56	103
Marginal column totals	52	60	112

Statistic = 0.33, parameter = 1, $P = 0.57$, sample estimates: non-constant unconditional function index = 0.05. Due to small sample sizes, several classes were merged to form the 'Other classes' category: Actinopterygii, Amphibians, Malacostraca, Mammals and Reptiles.

Table S10. Contingency table (unconditional exact test) showing the number of animal welfare impacts associated with ant invasions, as distributed by their severity and mechanism.

	Predation	Other mechanisms	Marginal row totals
Less severe	2	7	9
More severe	93	10	103
Marginal column totals	95	17	112

Statistic = 15.34, parameter = 1, $P < 0.001$, sample estimates: non-constant unconditional function index = 0.37. Due to small sample sizes, several mechanisms were merged to form the 'Other mechanisms' category: Competition, Disturbance and Poisoning / toxicity.

Table S11. Contingency table (unconditional exact test) showing the number of animal welfare impacts associated with ant invasions, as distributed by their severity and location of impact (island or other location).

	Island	Other location	Marginal row totals
Less severe	4	5	9
More severe	36	67	103
Marginal column totals	40	72	112

Statistic = 0.3, parameter = 1, $P = 0.59$, sample estimates: non-constant unconditional function index = 0.05.

References

1. Blackburn, T. M. *et al.* A unified classification of alien species based on the magnitude of their environmental impacts. *PLoS Biol* **12**, e1001850 (2014).
2. Mellor, D. J. *et al.* The 2020 Five Domains Model: including human–animal interactions in assessments of animal welfare. *Animals* **10**, 1870 (2020).
3. World Organisation for Animal Health (WOAH). *Animal welfare.* https://www.woah.org/en/what-we-do/animal-health-and-welfare/animal-welfare/. (2025).
4. IUCN. *IUCN EICAT Categories and Criteria. The Environmental Impact Classification for Alien Taxa (EICAT). First Edition.* https://iucn.org/resources/publication/iucn-eicat-categories-and-criteria-first-edition (2020).
5. Scottish Animal Welfare Commission. *Scottish Animal Welfare Commission: statement on animal sentience.* https://www.gov.scot/publications/scottish-animal-welfare-commission-statement-on-animal-sentience/. (2025).
6. *Animal Welfare (Sentience) Act 2022*. https://www.legislation.gov.uk/ukpga/2022/22#:~:text=2022%20CHAPTER%2022,of%20animals%20as%20sentient%20beings. (2022).
7. Indigo, N., Smith, J., Webb, J. K. & Phillips, B. Not such silly sausages: evidence suggests northern quolls exhibit aversion to toads after training with toad sausages. *Austral Ecol* **43**, 592–601 (2018).
8. Smith, K. G. *et al. A manual for the management of vertebrate invasive alien species of Union concern, incorporating animal welfare. 1st Edition. Technical report prepared for the European Commission within the framework of the contract no. 07.027746/2019/812504/SER/ENV.D.2.* (2022).
9. Feio, M. J. *et al.* The impacts of alien species on river bioassessment. *J Environ Manage* **374**, 123874 (2025).
10. Tyrrell, E. A. *et al.* Wildfire immediately reduces nest and adult survival of greater sage-grouse. *Sci Rep* **13**, 10970 (2023).
11. Carter, E. T., Eads, B. C., Ravesi, M. J. & Kingsbury, B. A. Exotic invasive plants alter thermal regimes: implications for management using a case study of a native ectotherm. *Funct Ecol* **29**, 683–693 (2015).
12. Parkes, J. P. *et al.* Rapid eradication of feral pigs (Sus scrofa) from Santa Cruz Island, California. *Biol Conserv* **143**, 634–641 (2010).
13. Jessop, T. S., Anson, J. R., Narayan, E. & Lockwood, T. An introduced competitor elevates corticosterone responses of a native lizard (Varanus varius). *Physiological and Biochemical Zoology* **88**, 237–245 (2015).
14. Dilley, B. J., Schoombie, S., Schoombie, J. & Ryan, P. G. 'Scalping' of albatross fledglings by introduced mice spreads rapidly at Marion Island. *Antarct Sci* **28**, 73–80 (2016).

15. Ley, D. H., Hawley, D. M., Geary, S. J. & Dhondt, A. A. House finch (Haemorhous mexicanus) conjunctivitis, and Mycoplasma spp. isolated from North American wild birds, 1994–2015. *J Wildl Dis* **52**, 669–673 (2016).
16. Deák, G., Holban, E., Sadîca, I. & Jawdhari, A. Sturgeon parasites: a review of their diversity and distribution. *Diversity* **16**, 163 (2024).
17. Gerlach, J. Impact of the invasive crazy ant Anoplolepis gracilipes on Bird Island, Seychelles. *J Insect Conserv* **8**, 15–25 (2004).
18. Hickman, C. R. & Watling, J. I. Leachates from an invasive shrub causes risk-prone behavior in a larval amphibian. *Behavioral Ecology* **25**, 300–305 (2014).
19. Molvar, E. M., Rosentreter, R., Mansfield, D. & Anderson, G. M. *Cheatgrass Invasions: History, Causes, Consequences, and Solutions. Hailey, ID: Western Watersheds Project, 128 pp.* https://westernwatersheds.org/wp-content/uploads/2024/02/Cheatgrass-Literature-Review-final.pdf (2024).
20. Wilson, J. R. U. *et al.* Frameworks used in invasion science: progress and prospects. *NeoBiota* **62**, 1–30 (2020).
21. Paul, E. S. & Mendl, M. T. Animal emotion: Descriptive and prescriptive definitions and their implications for a comparative perspective. *Appl Anim Behav Sci* **205**, 202–209 (2018).
22. Sözmen, B. I. Harm in the wild: Facing non-human suffering in nature. *Ethical Theory and Moral Practice* **16**, 1075–1088 (2013).
23. Mendl, M. T., Paul, E. S. & Mason, G. J. Animal welfare, affective states, and the Other Minds Problem (in press, 2026). In *Assessment of Animal Welfare. A Guide to the Valid Use of Indicators of Affective States. UFAW Animal Welfare Series* (Eds. Mason, G. J., Nielsen, B. L. & Mendl, M. T.) (John Wiley & Sons, Oxford).
24. Mazzamuto, M. V., Bisi, F., Wauters, L. A., Preatoni, D. G. & Martinoli, A. Interspecific competition between alien Pallas's squirrels and Eurasian red squirrels reduces density of the native species. *Biol Invasions* **19**, 723–735 (2017).
25. Frasca Jr, S. *et al.* Mycoplasmal conjunctivitis in a European starling. *J Wildl Dis* **2**, 336–339 (1997).
26. Animal Emergency Service. *Cane toads and dogs (everything you need to know about toad poisoning).* https://animalemergencyservice.com.au/blog/cane-toads-and-dogs/. (2015).
27. Nugent, G., Buddle, B. M. & Knowles, G. Epidemiology and control of Mycobacterium bovis infection in brushtail possums (Trichosurus vulpecula), the primary wildlife host of bovine tuberculosis in New Zealand. *N Z Vet J* **63**, 28–41 (2015).
28. Johnson, J. H. & Jensen, J. M. Hepatotoxicity and secondary photosensitization in a red kangaroo (Megaleia rufus) due to ingestion of Lantana camara. *Journal of Zoo and Wildlife Medicine* **29**, 203–207 (1998).
29. Ramírez-Cruz, G. A. *et al.* This town ain't big enough for both of us…or is it? Spatial co-occurrence between exotic and native species in an urban reserve. *PLoS One* **14**, e0211050 (2019).

30. Elsey, R. M., Mouton, E. C. & Kinler, N. Effects of Feral Swine (Sus scrofa) on Alligator (Alligator mississippiensis) nests in Louisiana. *Southeastern Naturalist* **11**, 205–218 (2012).
31. McClure, K. M., Fleischer, R. C. & Kilpatrick, A. M. The role of native and introduced birds in transmission of avian malaria in Hawaii. *Ecology* **101**, e03038 (2020).
32. Lai, L.-C., Hua, K.-H. & Wu, W.-J. Intraspecific and interspecific aggressive interactions between two species of fire ants, Solenopsis geminata and S. invicta (Hymenoptera: Formicidae), in Taiwan. *J Asia Pac Entomol* **18**, 93–98 (2015).
33. Schmidt, J. O. Pain and lethality induced by insect stings: An exploratory and correlational study. *Toxins* **11**, (2019).
34. Smith, J. E., Whelan, C. J., Taylor, S. J., Denight, M. L. & Stake, M. M. Novel predator–prey interactions: is resistance futile? *Evol Ecol Res* **9**, 433–446 (2007).
35. Wetterer, J. K., Wood, L. D., Johnson, C., Krahe, H. & Fitchett, S. Predaceous ants, beach replenishment, and nest placement by sea turtles. *Environ Entomol* **36**, 1084–1091 (2007).
36. Common, L. K., Sumasgutner, P., Dudaniec, R. Y., Colombelli-Négrel, D. & Kleindorfer, S. Avian vampire fly (Philornis downsi) mortality differs across Darwin's finch host species. *Sci Rep* **11**, 15832 (2021).
37. Evans, T. Quantifying the global threat to native birds from predation by non-native birds on small islands. *Conserv Biol* **35**, 1268–1277 (2021).
38. Biben, M. Predation and predatory play behaviour of domestic cats. *Anim Behav* **27**, 81–94 (1979).
39. Allen, B. L. *et al.* Animal welfare considerations for using large carnivores and guardian dogs as vertebrate biocontrol tools against other animals. *Biol Conserv* **232**, 258–270 (2019).
40. Haythorpe, K. *Competitive Behaviour in Common Mynas. An Investigation into Potential Impacts on Native Fauna and Solutions for Management. [Doctoral Thesis, Charles Sturt University].* https://researchoutput.csu.edu.au/en/publications/competitive-behaviour-in-common-mynas-an-investigation-into-poten-3. (2013).
41. Strubbe, D. & Matthysen, E. Experimental evidence for nest-site competition between invasive ring-necked parakeets (Psittacula krameri) and native nuthatches (Sitta europaea). *Biol Conserv* **142**, 1588–1594 (2009).
42. Feare, C. Ants take over from rats on Bird Island, Seychelles. *Bird Conserv Int* **9**, 95–96 (1999).
43. Chinchio, E. *et al.* Invasive alien species and disease risk: An open challenge in public and animal health. *PLoS Pathog* **16**, e1008922- (2020).
44. Allen, C. R., Epperson, D. M. & Garmestani, A. S. Red imported fire ant impacts on wildlife: a decade of research. *Am Midl Nat* **152**, 88–103 (2004).
45. Holway, D. A., Lach, L., Suarez, A. V, Tsutsui, N. D. & Case, T. J. The causes and consequences of ant invasions. *Annu Rev Ecol Syst* **33**, 181–233 (2002).
46. D'Alessio, R. M., Mc Aloon, C. G., Correia-Gomes, C., Hanlon, A. & O'Driscoll, K. Evaluation of a scheme to identify risks for tail biting in pigs. *PLoS One* **19**, e0305960 (2024).

47. Evans, T., Kumschick, S., Şekercioğlu, Ç. H. & Blackburn, T. M. Identifying the factors that determine the severity and type of alien bird impacts. *Divers Distrib* **24**, 800–810 (2018).
48. Harvey, A. M., Beausoleil, N. J., Ramp, D. & Mellor, D. J. A ten-stage protocol for assessing the welfare of individual non-captive wild animals: Free-roaming horses (Equus ferus caballus) as an example. *Animals* **10**, 148 (2020).
49. Mason, G. J. & Mendl, M. T. (in press, 2026). Measuring the unmeasurable: the construct validation of indicators of affect and welfare (In press). In *Assessment of Animal Welfare. A Guide to the Valid Use of Indicators of Affective States. UFAW Animal Welfare Series* (Eds. Mason, G. J., Nielson, B. L. & Mendl, M. T.) (John Wiley & Sons, Oxford).
50. Roche, J. R. *et al.* Invited review: Body condition score and its association with dairy cow productivity, health, and welfare. *J Dairy Sci* **92**, 5769–5801 (2009).
51. Harris, S., Shallcrass, M. & Cohen, S. A review of animal-based welfare indicators for calves and cattle. *Ruminants* **4**, (2024).
52. Kaur, M., Aggarwal, N. K., Kumar, V. & Dhiman, R. Effects and management of Parthenium hysterophorus: a weed of global significance. *Int Sch Res Notices* **368647**, 1–12 (2014).
53. Beaulieu, M. Capturing wild animal welfare: a physiological perspective. *Biological Reviews* **99**, 1–22 (2023).
54. Santicchia, F. *et al.* Stress in biological invasions: Introduced invasive grey squirrels increase physiological stress in native Eurasian red squirrels. *Journal of Animal Ecology* **87**, 1342–1352 (2018).
55. Narayan, E. J., Jessop, T. S. & Hero, J.-M. Invasive cane toad triggers chronic physiological stress and decreased reproductive success in an island endemic. *Funct Ecol* **29**, 1435–1444 (2015).
56. Evans, T. & Mendl, M. AWICIS Assessment Template. https://doi.org/10.6084/m9.figshare.29651189.v2 (2026) doi:10.6084/m9.figshare.29651189.v2.
57. Evans, T. AWICIS Test Assessments (x3). https://doi.org/10.6084/m9.figshare.29994313.v1 (2025) doi:10.6084/m9.figshare.29994313.v1.
58. Zhong, H. & Song, M. A fast exact functional test for directional association and cancer biology applications. *IEEE/ACM Trans Comput Biol Bioinform* **16**, 818–826 (2019).
