# Supplementary material for: Quantifying and categorising the animal welfare impacts caused by biological invasions
Source: Nat Commun. 2026 May 5;17:3899. doi: 10.1038/s41467-026-72154-9 (PMC13144615; doi:10.1038/s41467-026-72154-9)
Supplement: Supplementary file 2 — Reporting Summary [file 41467_2026_72154_MOESM2_ESM.pdf]

nature portfolio

Corresponding author(s): Thomas Evans

Last updated by author(s): Aug 31, 2025

Reporting Summary

Nature Portfolio wishes to improve the reproducibility of the work that we publish. This form provides structure for consistency and transparency in reporting. For further information on Nature Portfolio policies, see our Editorial Policies and the Editorial Policy Checklist.

Statistics

For all statistical analyses, confirm that the following items are present in the figure legend, table legend, main text, or Methods section.

n/a	Confirmed	
☐	☒	The exact sample size (n) for each experimental group/condition, given as a discrete number and unit of measurement
☒	☐	A statement on whether measurements were taken from distinct samples or whether the same sample was measured repeatedly
☐	☒	The statistical test(s) used AND whether they are one- or two-sided<br>*Only common tests should be described solely by name; describe more complex techniques in the Methods section.*
☒	☐	A description of all covariates tested
☒	☐	A description of any assumptions or corrections, such as tests of normality and adjustment for multiple comparisons
☐	☒	A full description of the statistical parameters including central tendency (e.g. means) or other basic estimates (e.g. regression coefficient) AND variation (e.g. standard deviation) or associated estimates of uncertainty (e.g. confidence intervals)
☐	☒	For null hypothesis testing, the test statistic (e.g. F, t, r) with confidence intervals, effect sizes, degrees of freedom and P value noted<br>*Give P values as exact values whenever suitable.*
☒	☐	For Bayesian analysis, information on the choice of priors and Markov chain Monte Carlo settings
☒	☐	For hierarchical and complex designs, identification of the appropriate level for tests and full reporting of outcomes
☒	☐	Estimates of effect sizes (e.g. Cohen's d, Pearson's r), indicating how they were calculated

Our web collection on statistics for biologists contains articles on many of the points above.

Software and code

Policy information about availability of computer code

Data collection	No custom code or software was used.
Data analysis	We analysed our data using contingency table tests (unconditional exact tests) using the FunChisq package in R (version 2025.05.1+513).

For manuscripts utilizing custom algorithms or software that are central to the research but not yet described in published literature, software must be made available to editors and reviewers. We strongly encourage code deposition in a community repository (e.g. GitHub). See the Nature Portfolio guidelines for submitting code & software for further information.

Data

Policy information about availability of data

All manuscripts must include a data availability statement. This statement should provide the following information, where applicable:

- Accession codes, unique identifiers, or web links for publicly available datasets
- A description of any restrictions on data availability
- For clinical datasets or third party data, please ensure that the statement adheres to our policy

The datasets generated in this study have been deposited in the Figshare database (https://doi.org/10.6084/m9.figshare.28553957.v2).

Research involving human participants, their data, or biological material

Policy information about studies with human participants or human data. See also policy information about sex, gender (identity/presentation), and sexual orientation and race, ethnicity and racism.

Reporting on sex and gender	Not applicable.
Reporting on race, ethnicity, or other socially relevant groupings	Not applicable.
Population characteristics	Not applicable.
Recruitment	Not applicable.
Ethics oversight	Not applicable.

Note that full information on the approval of the study protocol must also be provided in the manuscript.

Field-specific reporting

Please select the one below that is the best fit for your research. If you are not sure, read the appropriate sections before making your selection.

☐ Life sciences ☐ Behavioural & social sciences ☒ Ecological, evolutionary & environmental sciences

For a reference copy of the document with all sections, see nature.com/documents/nr-reporting-summary-flat.pdf

Ecological, evolutionary & environmental sciences study design

All studies must disclose on these points even when the disclosure is negative.

Study description	The study involved the development of a framework which can be used to assess changes to the welfare of animals that are caused by biological invasions. To test the framework, it was applied to global datasets of the biodiversity impacts associated with alien ant and alien bird invasions.
Research sample	The research sample consists of any animal (alien or native, wild or domesticated) that is affected by welfare impacts caused by alien ant and alien bird invasions. The scope of the study is global. As described in the methods, we selected alien ant and alien bird invasions with the aim being to obtain a broad range of welfare impacts caused by these widely separated taxonomic groups of alien species. We wanted to capture a broad range of impacts in order to test the framework's ability to assess them.
Sampling strategy	Our study was undertaken at the global scale, in order to capture as many welfare impacts as possible that were caused by alien ant and alien bird invasions.
Data collection	The data were collected through an online search carried out by T.E. The approach to this search is described in the methods, and all of the studies identified during this search are provided in the supporting information.
Timing and spatial scale	The spatial scale of the study was global. The search was conducted over several months in Autumn 2024.
Data exclusions	We excluded certain types of articles that we identified during the online search. We describe the rationale for their exclusion in the 'Data' section of the methods. We also include examples of excluded articles in the supporting information. We excluded data on impacts identified in artificial settings; we excluded data on impacts that were not published and peer reviewed; we excluded data on impacts that were caused by alien species in their native ranges (i.e., not as aliens).
Reproducibility	The methods adopted in the study are clearly described. The manuscript includes detailed guidelines on the ways in which the framework should be used. The methods for the online search are clearly described. The methods to analyse the data (contingency tables) are clearly described.
Randomization	Randomization is not relevant to this study, which assessed the published animal welfare impacts of biological invasions.
Blinding	Blinding is not relevant to this study, which assessed the published animal welfare impacts of biological invasions.

Did the study involve field work? ☐ Yes ☒ No

Reporting for specific materials, systems and methods

We require information from authors about some types of materials, experimental systems and methods used in many studies. Here, indicate whether each material, system or method listed is relevant to your study. If you are not sure if a list item applies to your research, read the appropriate section before selecting a response.

Materials & experimental systems

n/a	Involved in the study
☒	☐ Antibodies
☒	☐ Eukaryotic cell lines
☒	☐ Palaeontology and archaeology
☒	☐ Animals and other organisms
☒	☐ Clinical data
☒	☐ Dual use research of concern
☒	☐ Plants

Methods

n/a	Involved in the study
☒	☐ ChIP-seq
☒	☐ Flow cytometry
☒	☐ MRI-based neuroimaging

Plants

Seed stocks	Not applicable.
Novel plant genotypes	Not applicable.
Authentication	Not applicable.
